# The Potential Contribution of Biopolymeric Particles in Lung Tissue Regeneration of COVID-19 Patients

**DOI:** 10.3390/polym13224011

**Published:** 2021-11-20

**Authors:** Mohamed Abbas, Mohammed S. Alqahtani, Hussain M. Almohiy, Fawaz F. Alqahtani, Roaa Alhifzi, Layal K. Jambi

**Affiliations:** 1 Electrical Engineering Department, College of Engineering, King Khalid University, Abha 61421, Saudi Arabia; 2 Computers and Communications Department, College of Engineering, Delta University for Science and Technology, Gamasa 35712, Egypt; 3 Radiological Sciences Department, College of Applied Medical Sciences, King Khalid University, Abha 61421, Saudi Arabia; mosalqhtani@kku.edu.sa (M.S.A.); hmohiy@kku.edu.sa (H.M.A.); 438803534@kku.edu.sa (R.A.); 4 BioImaging Unit, Space Research Centre, Michael Atiyah Building, University of Leicester, Leicester LE1 7RH, UK; 5 Department of Radiological Sciences, College of Applied Medical Sciences, Najran University, Najran 1988, Saudi Arabia; ffalqahtani@nu.edu.sa; 6 Radiological Sciences Department, College of Applied Medical Sciences, King Saud University, P.O. Box 10219, Riyadh 11433, Saudi Arabia; ljambi@ksu.edu.sa

**Keywords:** COVID-19, tissue regeneration, lung, biopolymeric nanoparticles, stem cells, biopolymeric scaffolds

## Abstract

The lung is a vital organ that houses the alveoli, which is where gas exchange takes place. The COVID-19 illness attacks lung cells directly, creating significant inflammation and resulting in their inability to function. To return to the nature of their job, it may be essential to rejuvenate the afflicted lung cells. This is difficult because lung cells need a long time to rebuild and resume their function. Biopolymeric particles are the most effective means to transfer developing treatments to airway epithelial cells and then regenerate infected lung cells, which is one of the most significant symptoms connected with COVID-19. Delivering biocompatible and degradable natural biological materials, chemotherapeutic drugs, vaccines, proteins, antibodies, nucleic acids, and diagnostic agents are all examples of these molecules‘ usage. Furthermore, they are created by using several structural components, which allows them to effectively connect with these cells. We highlight their most recent uses in lung tissue regeneration in this review. These particles are classified into three groups: biopolymeric nanoparticles, biopolymeric stem cell materials, and biopolymeric scaffolds. The techniques and processes for regenerating lung tissue will be thoroughly explored.

## 1. Introduction

COVID-19, a rapidly evolving coronavirus, needs urgent therapy development. New immunosuppressive medications are urgently needed to preserve alveolar function and repair lung and systemic organ damage. Symptoms include scarring and airway obstruction. Despite extensive research on the causes of lung illness, no effective treatments have been identified. Early viral infection diagnosis also reduces long-term effects, including respiratory dysfunction. COVID-19‘s genetic structure, current transmission mechanisms and control strategies, etiology, clinical presentation, and lung effect have all been examined [1]. These exosomes generated by immunoregulatory DCs include an abundance of immunoregulatory proteins, compelling us to study their biodistribution to vital organs following intravenous injection [2]. Two new LNP formulations have been developed and evaluated for siRNA therapeutic delivery to the lungs, an organ severely damaged by SARS-CoV-2 infection. Injecting siRNA into these LNPs significantly reduced the viral load in the lungs and increased animal survival [3].

HPD/NPs have been used for nasal administration of inflamed lungs. In vitro and in vivo, HPD/NPs outperformed free HPD in terms of cellular absorption. In an inflammatory lung disease animal model, HPD/NPs lowered inflammatory cytokine levels and vascular permeability relative to free HPD [4]. Unlike monoclonal antibodies, ACE2‘s intrinsic catalytic activity for angiotensin II turnover may help reduce COVID-19 symptoms while protecting the lungs and cardiovascular system. Soluble ACE2 derivatives may therefore be employed as next-generation therapies to address pandemic and future epidemic demands [5]. Specific materials can be delivered directly to injured lung tissue through a catheter or aerosol. These include exosomes, microbubbles, adenosine nanoparticles, new bio-objects, direct aerosol targeted pulmonary administration, and catheter-based drug delivery [6]. Many off-label medications that have been licensed for other ailments are currently being studied in clinical trials. These MSCs have been examined in both animal and human models for the therapy of many pathologies, including acute and chronic lung diseases. ARDS, the most common complication, has demonstrated promising outcomes in treatment [7].

Hydration was changed to generate an aerosolized nanoliposomal carrier for AL-Rem. This study examined the stability and aerodynamic properties of liposomes produced by lung cancer cells. AL-Rem has a hydrodynamic diameter of 71.46 +/−1.35 nm and a surface charge of 32 mV. Intact A549 cell monolayers were protected by AL-Rem. In AL-Rem a continuous release profile was observed in simulated lung fluid, with full drug release 50 h later [8]. The pharmacologically active and lung-protective lisinopril molecule covalently bonded to L-PLGA was employed to encapsulate remdesivir. In addition, lisinopril and its intracellular targeting protein (RNA-dependent RNA polymerase) were confirmed using a binding model (RdRp) [9]. Viral receptors and entrance cofactors on host cell surfaces influence many viruses’ tissue tropism. An anti-NRP monoclonal antibody improves infectivity by increasing NRP1 binding to furin-cleaved substrates. One case of a mutant with a novel furin cleavage site had no need for NRP1 [10]. The present epidemic has spurred interest in the development of infected human tissues. When analyzing deep-tissue information, NIR-II imaging beats other methods [11]. 

The effects of ALA on the patients’ lungs and fat were investigated. Design, planning, and implementation of ALA effects on the patients’ respiratory systems and adipose tissue were studied. The data imply that alpha-linolenic acid protects the lungs. However, it may increase adipose tissue glucose transporter-4 gene expression while reducing the prognosis [12]. Using AI in outpatient treatment during a pandemic may help doctors notice overlooked signs and diagnose lung damage [13]. Infection with COVID-19 in the upper or lower respiratory tract may cause mild to severe respiratory disease by releasing pro-inflammatory cytokines such as IL-1b and IL-6. The inflammasome is triggered when there are any interactions with the toll-like receptor (TLR). Suppression of pro-inflammatory IL-1 and IL-6 family members may help cure inflammatory conditions like viral infections [14]. Despite the disease’s youth, lung CT imaging has been used to assess its pulmonary effects [15]. The most prevalent pathologic hallmark of the infected lung damage is acute and ordered alveolar disintegration. It may appear as a fibrinous or organized pneumonia. In instances of bacterial pneumonia, acute neutrophilic infiltrates have been described [16]. 

An unexpected COVID-19 infection with moderate-severe bilateral lung injury and minor desaturation was seen after bacterial superinfection. In addition, the patient was given corticosteroids and antivirals [17] because NETs are linked to tissue damage caused by sepsis. The presence of NETs in the patient’s lung tissue was studied. NETs were counted using CIF or MPO-DNA PicoGreen. COVID-19 NETs kill human lung epithelial cells (A549 cells) [18], thickening the lung alveolar wall and raising blood cytokine levels. Serum amyloid P (SAP) has been shown to relax the innate immune system in fibrosis models and clinical trials [19]. Some drugs, especially lopinavir and ritonavir, have been linked to serious liver damage. The infection can harm other organs due to viral infection, immune-mediated inflammation, or drug toxicity [20]. This review will examine and analyze the role of biopolymeric particles in COVID-19 patients’ lung healing. This study discusses biopolymeric nanoparticles, biopolymeric stem cell materials, and biopolymeric scaffolds. These will include the most advanced lung tissue regeneration techniques. Their regeneration success is also compared. 

**Figure 1:**
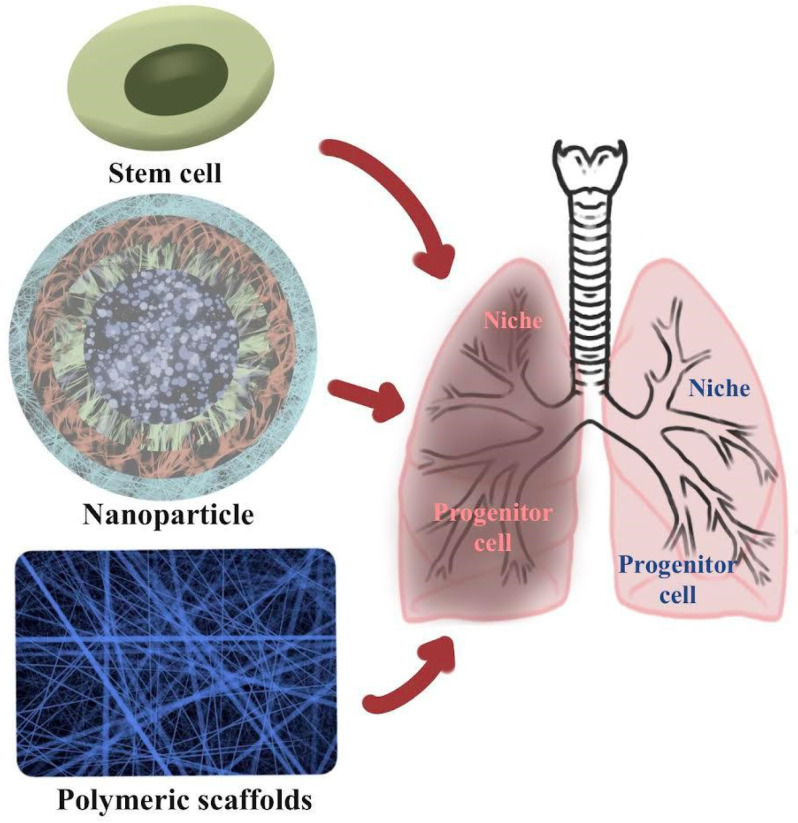
The effects of stem cells, nanoparticles, and biobiopolymeric scaffolds.

Figure 1 shows the effects of stem cells, nanoparticles, and biopolymeric scaffolds. Nanoparticle-based molecular imaging agents employ targeted agents to provide better insight into disease processes rather than just structural and functional information. As for COVID-19 lung injury treatments, stem cell therapies and their released EVs are gaining popularity. A medically and physiologically appropriate 3D printed scaffold composed of chitosan/polycaprolactone bioink has been created for lung tissue engineering.

## 2. Regeneration of Lung Tissues Using Biopolymeric Nanoparticles for COVID-19 Patients

**Figure 2:**
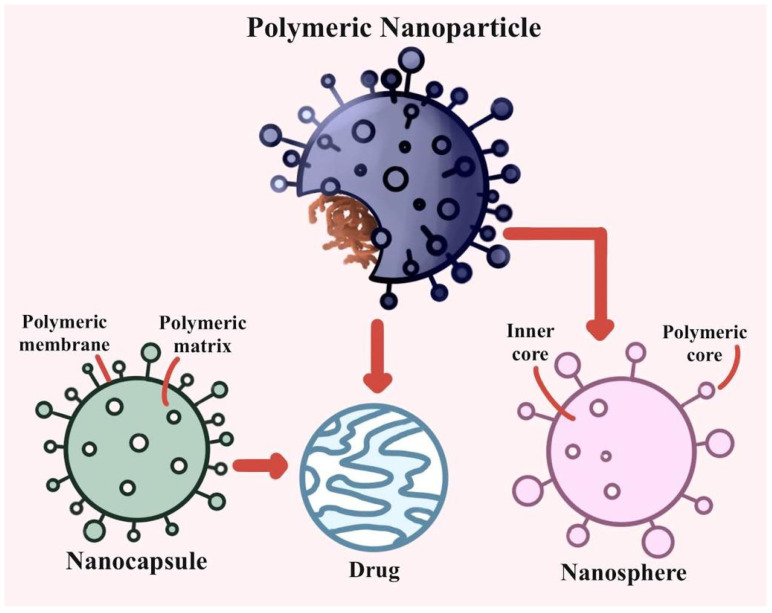
Different types of biopolymeric nanoparticles.

Multiplexed point-of-care devices will allow for quick genotyping and biomarker evaluation, allowing for therapy optimization and customization for each patient. The transport of nanoparticle-encapsulated medications to the site of action would be improved if the effective concentration was increased while the systemic dosage and side effects were reduced. Controlled and tailored medication release from polymers will enhance pharmacokinetics and bioavailability. By using the body’s natural healing mechanisms [21], nanotechnology will make it simpler to produce better tissue implants in vitro and scaffolds to promote regeneration in vivo. Using CT lung images, a machine learning system can identify ill and healthy lungs. This step reduces the effect of intensity fluctuations and noise between CT slices. The CT lung image backdrop is then isolated using thresholding and morphological approaches. Each dataset is subjected to GLCM and an optimization wrapper technique for texture-based feature extraction. The gathered properties are classified using a deep convolutional neural network [22]. Biopolymeric nanoparticles may be used therapeutically because of their low cytotoxicity, biodegradability, bioavailability, biocompatibility, and accurate delivery to the target site of action. They may be used to administer therapeutic drugs and promote tissue development in inflammatory organs such as the lungs [23]. The interaction between coronavirus spike and hACE2 proteins with and without nanoclays has been simulated and the affinity and cohesiveness of the SARS-CoV-2 spike and nanoclays compared [24]. A therapeutic notion of introducing nanoparticles to embryonic airways and later removing them was investigated by putting nanoparticles into a pregnant pig’s trachea. All lung lobes received 3 mL of injection volume; no lung damage was detected [25]. Figure 2 shows the different types of biopolymeric nanoparticles.

IPF is a deadly illness that may appear in many ways. Inefficient medication delivery to the lungs and “damaged” type II AEC II have hampered IPF therapy. On the other hand, MOMC-per-NPs (per-NPs) combines the two. MOMCs have many PER NPs on their surface that target the lungs after interacting with MMP-2 in IPF tissues [26]. The deterioration of the hydrogels was assessed utilizing a simple and non-invasive in vivo hydrogel tracking methodology employing UCNPs. For monitoring hydrogel deterioration, the UCNPs’ fluorescence intensity change and hydrogel weight change were extremely like those of FITC covalently attached hydrogels [27]. Through physical adsorption, photocrosslinkable thermoresponsive polymer sheets were generated.

Films at temperatures above and below the polymer’s LCSTs have been adapted to see how the adsorption preparation temperature affects the final film. This allows CoV-2 to enter host cells by attaching to ACE2 receptors [28]. The nanotraps blocked SARS-CoV-2 infection in host cells by preventing the binding of the SARS-spike CoV-2 protein with the ACE2 protein. At least two anti-SARS-CoV-2 neutralizing antibodies and phagocytosis-specific phosphatidylserines were added to the liposomal-based nanotrap surfaces [29]. A/H1N1, SARS-CoV-2, and MERS-CoV are some of the new emergent infectious illnesses for which nanomedicine is being employed [30]. RepRNA-CoV2S, an alphavirus-derived replicon RNA vaccine candidate, encodes the SARS-CoV-2 spike (S). It was shown that LIONs improved vaccine stability, delivery, and immunogenicity. The LION/repRNA-CoV2S vaccination elicited anti-SARS-CoV-2 S protein IgG antibody isotypes suggestive of a type 1 T helper cell response in mice [31]. The optimization, structure, purity, and shape of ZnO nanoparticles were examined. In silico, the interaction of ZnO NPs with COVID-19 targets such as the ACE2 receptor, COVID-19 RNA-dependent RNA polymerase, and major protease was investigated. A study on ZnO NPs internalization used human lung fibroblast cells [32]. These cells endocytose SARS-CoV-2 spike protein nanoparticle virosomes. TW-37, a novel KIM-1-mediated endocytosis inhibitor, and anti-Kim-1 antibodies both reduced absorptions. Higher KIM-1 expression facilitated virosome absorption in human renal tubuloids. In KIM-1 positive cells, the SARS-CoV-2 receptor ACE2 was reduced [33]. Despite the disease’s widespread prevalence, no vaccinations or treatments exist. ENPs are exosome-like vesicles produced by plants. Molecular docking experiments looked at the potential of SSRIs to interact with the primary protease of the coronavirus that causes SARS-CoV-2 [34].

To boost the medicine’s efficacy, LPH nanoparticles were encapsulated. Study participants included FH, paroxetine, nisoxteine, repoxteine RR, and repoxteine SS. To avoid viral infection, synthetic mRNA was used to make a soluble version of hACE2. Researchers employed novel LNPs to transfect mammalian cells and bundle mRNA [35]. An intravenous LNP injection delivered the mRNA to the liver. hsACE2 levels peaked at six hours and then steadily declined over the next few days. This suggests that SARS-CoV-2 pathogenesis occurs in the lungs, where researchers found hsACE2 in the bronchoalveolar lavage fluid after injecting LNPs into the lungs for 24 h. Co-immunoprecipitation shows mRNA makes hsACE2 [36]. Vaccine-induced allergy responses are quite uncommon. BNT162b2 is an mRNA vaccine encapsulated in lipid nanoparticles and coupled with additional components to allow cell entry. Subjects who had a “history of a major adverse response to the vaccination and/or a serious allergic reaction to a component of the study medicine” were excluded from the pivotal phase III clinical trial [37]. Zofin (previously Organicell Flow) is manufactured from human amniotic fluid soluble and nanoparticle components (extracellular vesicles and exosomes). The clinical outcomes of Zofin treatment in three critically ill patients with COVID-19 infection are presented below. There was no difference between the COVID-19 patients and those with respiratory failure [38]. The toxicity of anti-CoV NPs has been studied on numerous levels. It addresses nano–bio interactions, in vitro (lung cells) and in vivo assessments, as well as human ramifications [39].

Two of the most used coronavirus vaccines were being developed on a novel platform based on mRNA containing LNPs in 2019. Clinical studies and continuing vaccinations have shown that they provide very high levels of protection with variable degrees of adverse effects. The nature of the claimed detrimental effects, on the other hand, is still unclear. LNPs, which have been utilized in several preclinical investigations, cause a lot of inflammation in mice. Massive neutrophil infiltration, activation of different inflammatory pathways, and generation of many inflammatory cytokines and chemokines were all seen after intradermal injection of these LNPs. The same dosage of LNP given intranasally caused comparable inflammatory reactions in the lungs as well as a significant death rate [40]. The ACE2 receptor’s spike protein, helicase, and protease sites showed a high affinity for flavonoid-based substances, which is exploited by the COVID-19 coronavirus to infect cells and produce COVID-19. In vitro studies have shown that caflanone inhibits viral entry factors including ABL-2, cathepsin L, cytokines (IL-1beta, IL-6, IL-8, Mip-1alpha, TNF-alpha), and PI4Kiiibeta, as well as AXL-2, which aids coronavirus transmission from mother to fetus.

To overcome bioavailability limitations and enhance therapeutic effectiveness, smart drug delivery techniques such as nanoparticle drones loaded with these phytomedicines have been explored [41]. The ability of AlphaMSH to target nanoliposomes to IBD locations has been investigated. AlphaMSH is an endogenous tridecapeptide that interacts with the melanocortin-1 receptor and has anti-inflammatory and immunomodulatory properties. The ability of a liposomal nanoparticle carrying alphaMSH to preferentially target inflamed intestines has been evaluated [42]. Because of the high degree of vascularization across a wide surface area, the pulmonary route has numerous advantages, including fast absorption and effective avoidance of first-pass metabolism. Aerosolization of NPs is now being studied in detail and has great potential for the targeted delivery of therapeutic substances for a variety of diseases. To move through the respiratory tract and overcome the obstacles posed by the pulmonary system, NPs must possess specific properties [43]. A potent two-stage neutralization method was utilized to create a decoy nanoparticle against COVID-19: viral neutralization in the first phase, followed by cytokine neutralization in the second. The nanodecoy is produced by combining cellular membrane nanovesicles synthesized by human monocytes with genetically modified cells that are stable in expressing ACE2 receptors and have the same antigenic exterior as donor cells. These nanodecoys successfully protect host cells against infection by pseudoviruses and genuine viruses by competing for viral contact with host cells. SARS is caused by the virus SARS-CoV-2 [44].

Existing antiviral therapies suffer from a variety of disadvantages, including drug resistance and the inability to target viral proteins, necessitating the development of new nanotherapeutic or nanovaccine methods. Due to their unique physical and biological characteristics, nanoparticles have the potential for both antiviral therapy and vaccinations against viral infections. Aside from the physiological characteristics of the respiratory system, there is an urgent need for nano-designs in the manufacture of vaccinations and antiviral medicines for airway-localized delivery [45]. In an in vitro environment that mimicked deep lung airways, nanoparticles were created and evaluated in an in vitro environment. A fluorescent live-dead test was used to assess cellular damage. The morphology of the cells was examined using AFM. The expression of inflammatory markers, particularly IL6 and CCL2, was measured by qRT-PCR.

**Figure 3:**
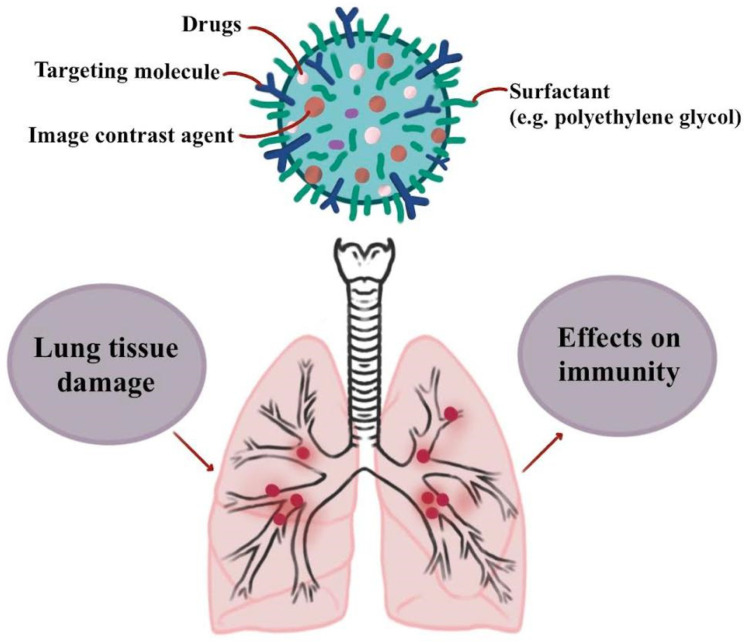
The effects of nanoparticle drugs on immunity.

An ELISA was used to evaluate IL6 and validate qRT-PCR findings at the protein level. Finally, the levels of reactive ROS in each group were assessed. AFM studies showed that NO exposure alters cell shape before and after shear exposure, and qRT-PCR revealed a significant decrease in IL6 and CCL2 production when the cells were treated with NO through nanoparticles [46]. Statistical shape modeling was used to create four diseased lung models with different degrees of bronchial dilation and constriction in the left-lower lobe. A low Reynolds number k-omega turbulence model and a Lagrangian tracking method were used to simulate respiratory airflow and particle deposition. Figure 3 shows the effects of nanoparticle drugs on immunity.

The flow partitions between the left and right lungs, as well as the lower and higher lobes of the left lung, varied tenfold between the most dilated and restricted models, suggesting significant differences. Much lower dosages were anticipated on the surface of the restricted left-lower bronchioles G4–G9, as well as in the peripheral airways beyond G9 of the left-lower lung [47]. Nanocurcumin was studied for its therapeutic effects on the quantity and reactivity of Th17 cells in COVID-19 patients with moderate and severe illness. The study included 40 patients in an intensive care unit who had moderate COVID-19 and 40 patients who had severe COVID-19; serum cytokine levels were all measured before and after treatment in both the nanocurcumin and placebo-treated groups [48]. Interaction of magnetic IONPs (Fe_2_O_3_ and Fe_3_O_4_) with the SARS-CoV-2 spike protein receptor binding domain S1-RBD, which is essential for viral attachment to host cell receptors, was investigated in docking research [49]. Antiviral medicines have been proven to enhance bioavailability, regulate release, and decrease adverse effects when encapsulated in NPs. Quantitative data on how virally infected cells absorb NPs is restricted as compared to uninfected cells. The absorption of fluorescently tagged polymer NPs in RV infected cells was studied. Several RV infection MOI and NP uptake regimens were examined by the researchers. Although this was not the case in all instances, RV infection did result in a substantial increase in NP absorption in some cases [50].

Leukosomes are leukocyte-derived nanovesicles that may be utilized to target inflamed vasculature in vivo, which has been linked to diseases including cancer, cardiovascular disease, and sepsis. Researchers investigated the anti-inflammatory properties of leukosomes in a lethal model of LPS-induced endotoxemia, which matched the cytokine storm seen in COVID-19 infection after encapsulating dexamethasone [51]. Significantly, NASAR mRNAs supplied by lipid-derived TT3 nanoparticles enhance the expression of putative SARS-CoV-2 antigens. The FDA-approved lipid nanoparticle compound MC3 elicits almost two orders of magnitude more antigen-specific antibodies in vaccinated mice than TT3 nanoparticles and NASAR mRNAs. NASAR mRNAs should be investigated further as SARS-CoV-2 vaccine candidates [52]. As the current COVID-19 epidemic has shown, the impact of airborne NPs on human health is a hot study topic in in vitro models for such research [53].

Existing membranes have low bioactivity and regeneration capacity. A flexible poly (capro-lactone) composite membrane incorporating ZnO nanoparticles is designed to overcome these issues. Electrospinning of PCL and ZnO particles creates the membranes. The modified membrane’s physical, mechanical, and in vitro degrading properties are explored in depth. In vitro studies are also being conducted on its osteoconductivity and antimicrobial activities [54]. Cationic polymers and LNPs are employed to carry plasmid DNA into the nucleus of host cells in lung tissues, where it promotes a particular immune response. In human testing [55], NVX-CoV2373 is a recombinant protein nanotech vaccine. In a mouse model of acute lung damage, hAFS cells were tagged with dual polymer-coated nanoparticles. HAFS cells migrate to the lung, utilizing extremely sensitive in vivo imaging [56]. AgNP dressings are often used to treat acute and chronic wounds. However, whether AgNP is harmful to wound healing is unknown. To show how AgNP speeds up wound healing, the speed of wound healing was measured using zebrafish fin regeneration. This approach also allowed for in vivo evaluation of AgNPs’ detrimental effects on wound healing [57]. AuNPs have the potential to accelerate wound healing by stimulating tissue regeneration, connective tissue development, and angiogenesis [58]. NPs may enter the body via the lungs, causing harmful consequences. Despite their widespread use, the health implications of NP exposure remain unknown. Concerns about human health are addressed by examining the biophysical features of naturally occurring and synthesized NPs [59]. To anticipate cell behavior, HSP27 and SAPK/JNK proteins surfaced as promising indicators of intracellular stress and susceptibility to endogenous redox alterations, respectively [60]. Time-of-flight inorganic and organic compounds can be detected directly using ToF-SIMS, but their capacity to identify nanoparticles in tissue sections is yet unknown. Comparative investigations with known nanoparticle detection methods may help quantify this technique’s usefulness for nanotoxicological problems [61]. Using the same nanoparticle samples, inhalation and intratracheal administration tests were conducted to compare the similarities and differences in lung tissue between the two investigations [62]. The transformation of hyaluronic acid into nanoparticles changed its physiological characteristics and made it resistant to ionizing radiation. They had a significant impact on cytokine production, particularly in the pro-fibrotic signaling pathway, in lung tissue [63].

## 3. Biopolymeric Materials of Stem Cells for Lung Tissue Regeneration

COVID-19 is being treated with antiviral, antimalarial, and anti-inflammatory medicines. While these therapies may help patients recover and live longer, they may not fully heal the disease’s lung damage. Due to the EVs’ immunomodulatory, antioxidant, and reparative therapeutic activities, those utilizing COVID-19 alone or in conjunction with other treatments may benefit [64]. SARS Coronavirus 2 (SARS-CoV-2) is a coronaviridae virus that causes respiratory difficulties. A cytokine storm happens when an infection produces many inflammatory cytokines. These cytokines may cause inflammation-induced lung damage and even death. One treatment option for ARDS is an MSC transplant (MSC). By immunomodulating, MSCs control inflammation and reduce lung damage. Lung cell death has also been found to be prevented and repaired by MSCs [65]. Although only a small percentage of COVID-19 patients reach a critical stage, the high mortality rate and large number of cases inflict a significant financial burden on society and create unprecedented demands on healthcare resources. Even while ARDS primarily affects the lungs, many patients have multiorgan failure. Regrettably, there is no conclusive or curative medication for this condition, so new medicines are urgently needed. Preclinical and early clinical evidence demonstrates that MSC treatment may repair and restore damaged tissues and organs [66].

**Figure 4:**
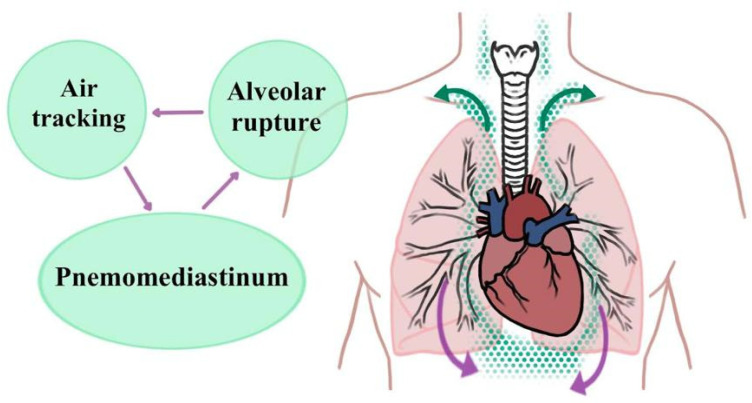
Extending beyond the pulmonary hilum.

A cytokine storm in the lungs causes pathological lesions, pulmonary edema, and acute respiratory distress syndrome (ARDS), all of which may be fatal. Current therapies do not address both the cytokine storm and tissue healing. MSCs are anti-inflammatory, immunomodulatory, and may promote endogenous regeneration. So, MSC therapy may be a COVID-19 treatment option. When administered intravenously to COVID-19 patients, clinical-grade MSCs have been shown to improve immune function and lung function [67]. The ability of SCs to regenerate and mend human tissues opens new therapeutic possibilities. Some downsides of SC treatment include patient biocompatibility and the danger of cross-infection. To overcome these barriers, nanoconjugates are added to SCs, which will help cure COVID-19 [68]. The use of poorly defined MSC products increases the risk of thromboembolism. It is necessary to reach an agreement on registered clinical trials [69]. Primarily, the virus affects cells that line the airways and alveoli. Mechanized breathing may exacerbate epithelial damage. Acutely infected patients who survive the acute phase of infection are at risk of acquiring lung diseases connected to various stem/progenitor cells [70]. Figure 4 shows these extending beyond the pulmonary hilum.

Patients who are old or who have medical comorbidities are more likely to acquire a severe illness. Hypoxia, respiratory discomfort, and lung injury may all be caused by inflammation, pulmonary edema, and an over-reactive immune response. MSCs are immunomodulators with a lot of potency. Many in vivo animal studies and ex vivo human lung models have shown the MSCs’ remarkable ability to prevent lung injury, decrease inflammation, suppress immunological responses, and assist in alveolar fluid evacuation. Antimicrobial and pain-relieving compounds are also produced by MSCs. The cells go straight to the lungs after an intravenous injection, where most of them remain sequestered, which is a significant advantage in the treatment of pulmonary illness [71]. Many human clinical investigations, including research into ARDS, have shown the in vivo safety of local and intravenous injection of MSCs.

**Figure 5:**
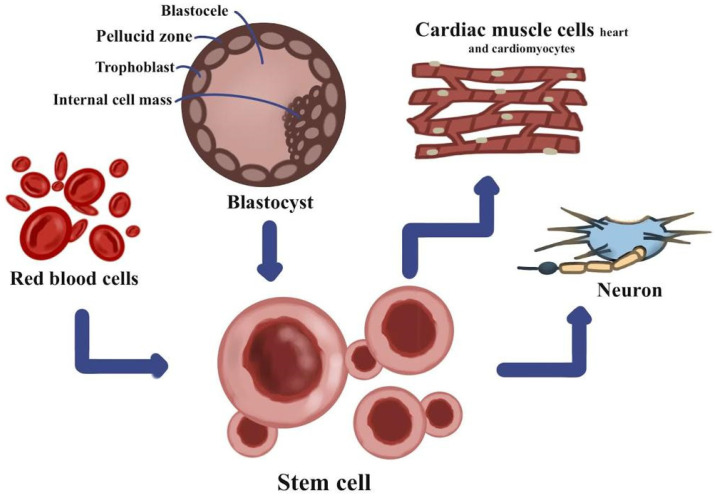
The stem cells in different cell types.

Because of the lack of treatment options for ARDS, the number of severe cases has skyrocketed across the globe. The major pathologic characteristics of severe or critical COVID-19 are like those of ALI/ARDS, which are characterized by cellular fibromyxoid exudates, significant pulmonary inflammation, pulmonary edema, and hyaline membrane development. MSCs have previously been shown to be useful in the treatment of both infectious and non-infectious forms of ALI/ARDS, suggesting that they might be used to treat COVID-19 [72]. At this moment, there are no treatments for SARS-CoV-2. The results of MSC treatment are excellent after SARS-CoV-2 infection since these cells decrease immune system overactivation, enhance endogenous healing, and improve the pulmonary microenvironment. A study was conducted on the potential applications of the most common MSCs, which are represented by ASCs in COVID-19 [73]. A COVID-19 infection may produce a wide range of symptoms, from asymptomatic infection to severe pneumonia and ARDS. SARS-CoV-2 mostly affects the lungs, which take a long time to repair. SARS-CoV-2 infects the lungs via angiotensin-converting enzyme 2 receptors, eliciting an immunological response that results in an influx of immunocompetent cells and a cytokine storm that damages and malfunctions target organs [74]. As MSCs have a well-established immunomodulatory function as an alternative therapy for the treatment of inflammatory and autoimmune disorders, in several investigations MSC-derived EVs such as exosomes have been shown to exhibit therapeutic effects comparable to those of parent MSCs [75]. Figure 5 shows the stem cells in different cell types.

Most of the clinical therapy is supportive and dependent on the patient’s immune response, which may result in a cytokine storm, lung edema, airway dysfunction, and ARDS, all of which can result in multiorgan failure and death. Human MSCs derived from a variety of tissues have shown promise in a variety of immunocompromised disorders, inhibiting immune system overactivity, and promoting endogenous repair by improving the microenvironment, resulting in an increase in MSC infusion demand in ICU patients with COVID-19-related ARDS. For those with severe COVID-19, MSC therapy is started right immediately [76].

The purpose of this study was to investigate the role of MSCs in COVID-19 treatment by reporting two confirmed COVID-19 patients in Wuhan. Immunological signs improved after MSC transplantation, but inflammatory markers decreased (interleukin-6 and C-reactive protein). A high-flow nasal cannula may be used as a first-line treatment for those with ARDS. Although SaO_2_ and partial pressure of PO_2_ increased following MSC transplantation, the fraction of FiO_2_ decreased [77]. ARDS is a life-threatening illness with a high mortality rate. The majority of those who have survived are living in deplorable conditions. The current clinical therapeutic choices include respiratory support and hydration restriction, with no prescription medication being advised. MSCs have been proposed as a possible lung disease treatment. In animal models of ARDS, MSCs have been demonstrated to create soluble, physiologically active molecules that have a range of protective advantages [78]. MSC therapy has emerged as a viable COVID-19 therapeutic option despite a scarcity of preclinical data.

The first preclinical study on MSC treatment for immune/inflammatory lung illnesses was published in 2003, and the first clinical usage of MSC therapy for graft versus host disease was described in 2004. Since these initial discoveries, preclinical research demonstrating the beneficial benefits of MSC immunomodulation has exploded, with immune/inflammatory diseases accounting for roughly a third of MSC therapy clinical trials [79]. LSCs that are proliferating and transdifferentiating may help in lung damage recovery. The distal airways of the lungs are innervated by the vagus nerve. Although the 7nAChR signaling pathway regulates lung infection and inflammation, it is unknown if it also regulates LSCs [80]. Both polarized macrophages (M2) and conventionally polarized macrophages suppressed SARS-CoV-2 infection (M1). Only M1 macrophages produced inflammatory chemicals such as IL-6 and IL-18, which inhibited lung cell growth and increased mortality.

When an ACE2 inhibitory antibody was used to limit viral entrance into target cells, the virus was nearly completely cleared, and lung cells were protected [81]. Adult lung regeneration strategies have been developed in vivo or ex vivo using endogenous stem cells or pluripotent stem cell derivatives due to a fundamental lack of understanding of the mechanisms that control human lung development, the precise identity and function of human lung stem and progenitor cell types, and the genetic and epigenetic control of human lung fate [82]. Allogenic bone marrow stem cells are used to isolate MSCs and their exosomes (MSCs-Exo) [83]. It is unknown if a single BASC can regenerate both the distal and alveolar airways in a bidirectional manner. Single BASCs evolve into club cells, ciliated cells, AT2 and AT1 cells, according to in vivo single cell clonal research [84].

Monolayers generated from ALOs, native airway cells, or hiPSC-derived alveolar type-II (AT2) pneumocytes were infected with SARS-CoV-2 to construct in vitro COVID-19 lung models. COVID-19 patient-derived respiratory samples from diverse cohorts were accurately recreated by infected ALO-monolayers. Both experiments were carried out in ALO monolayers with well-mixed proximodistal airway components [85]. Chronic viral infection requires the use of airway (proximal) cells, while terminal illness necessitated the use of distal alveolar differentiation (AT2AT1) cells to induce an unusually powerful host immune response. Lung fibrosis proceeds from the periphery to the center when Cdc42 activity in alveolar stem cells is reduced (AT2 cells). Cdc42-null is also a factor to consider. AT2 cells are unable to produce new alveoli in both post-pneumonectomy and untreated aged rats, placing them under mechanical stress. Mechanical strain activates a TGF signaling pathway in AT2 cells, hastening the progression of lung fibrosis from the periphery to the core. Poor alveolar regeneration, mechanical stress, and the development of lung fibrosis are all linked in our results [86]. Idiopathic pulmonary fibrosis is an interstitial lung disease characterized by alveolar remodeling and progressive pulmonary function loss due to chronic alveolar injury and an inability to repair the respiratory epithelium. AT1 and AT2 cells normally surround alveolar structures on histological analysis, however they have been replaced with fibrotic lesions and honeycomb structures expressing atypical proximal airway epithelial markers. Bronchial epithelial stem cells (BESCs) may form AT2 and AT1 cells, as well as honeycomb cysts, after bleomycin-induced lung injury [87]. Most people infected with the COVID-19 virus show mild to moderate symptoms and recover on their own within 14 to 20 days. However, 15% of people with the condition progress to later stages, with 2.5% dying as a result.

Patients with severe disorders are mostly older (65 and above) and have many comorbidities. During the incubation and non-severe phases, immune responses to the COVID-19 virus need early activation of a specific adaptive immune response to eliminate the virus and prevent it from escalating to severe stages. Those with a faulty bridge to adaptive immunity have a higher innate immune response due to a lack of adaptive immune cell input [88]. In AT2 in vitro human models, SARS-CoV-2 infection of the distal lung epithelium is reproduced. They are made up of genetically modified induced pluripotent stem cells that can develop at the air-liquid interface (iAT2s). Infected iAT2s produce cytokines through NF-kB target genes, have a delayed epithelial interferon response, and have lost the mature lung alveolar epithelial program [89]. Infected iAT2s exhibit cellular toxicity over time, as seen in COVID-19 lung autopsies, which may contribute to the loss of these crucial alveolar facultative progenitors. Based on the findings of our phase 1 trial [90], a phase 2 study has been conducted to determine the efficacy and safety of human UC-MSCs for treating severely affected COVID-19 patients with lung damage. As a biologic therapy for COVID-19, exosome extracellular vesicles are addressed in two ways. MSCs are being used more often in COVID-19 patients with a severe clinical cytokine storm and severe pneumonia [91].

This notion is supported by in vivo evidence of their multipotency and self-renewal [92]. Human lung organoids and bud tip progenitor organoids, for example, are not identical to human pluripotent stem cells [93]. Mesenchymal stem cells may produce tolerogenic dendritic cells, which can help the immune system grow [94]. Stem cells must develop in response to the tissue’s requirements to keep the lungs healthy. These findings could help researchers better understand how the lungs are maintained and lead to the development of new medications to boost endogenous regeneration in people with lung disease [95]. The numerous mechanisms of a healthy lung facilitate rapid healing and restoration of function in the case of minor acute damage. Researchers looked at all the typical endogenous pathways of lung growth, homeostasis maintenance, and repair for human therapeutic lung regeneration [96]. By increasing in vivo pluripotent stem cell generation and adult stem cell manipulation, this finding is predicted to change lung regenerative medicine [97]. Bioengineered lung and tracheal tissue might be a feasible alternative to organ/tissue transplantation, and stem cell therapy is a relatively new and commonly utilized therapeutic technique [98]. It is possible that using stem/progenitor cells as a therapeutic adjuvant might help those with severe lung injury [99]. Researchers are striving to convert pluripotent cells such as induced pluripotent stem cells and embryonic stem cells into differentiated adult lung cells to create disease-specific in vitro models and personalized therapeutics. To create distal lung epithelial cells from stem cell progenitors, researchers have utilized precise media, coculture with mesenchymal components, or treatments that simulate lung development [100]. MiR-27a-3p is released into alveolar macrophages by mesenchymal stem cells to prevent acute lung injury [101].

## 4. Regeneration of Lung Tissue Using Biopolymeric Scaffolds

Due to the significant morbidity and mortality associated with lung diseases, novel biomaterials and scaffolds are needed to stimulate lung tissue regeneration while also shielding it from inflammation and microbial assault. The potential of creating lung tissue using electrospun fiber meshes composed of P (VDF-TrFE) and ZnO has been investigated. Numerous fiber mesh textures (collected at 500 and 4000 rpm), as well as simple and composite compositions including ZnO/P (VDF-TrFE) ratios of (0/100) and (20/80), show anti-inflammatory, anti-bacterial, and mechano-electrical characteristics. The biological response of A549 alveolar epithelial cells to lung-infecting bacteria has been investigated, as well as the morphological, physicochemical, mechanical, and piezoelectric properties of the scaffolds. After 6 h, the ZnO in the composite scaffolds induced a strong anti-inflammatory response in A549 cells, as shown by a substantial decrease in IL-1, IL-6, and IL-8 production. TGF and the antimicrobial peptide HBD-2 were substantially increased in all scaffold types, but especially in aligned composite scaffolds [102].

The scaffolds were tested physiochemically, physiologically, and mechanically to determine their ability to support MRC-5 cell growth, proliferation, and migration. According to the results, the chitosan/polycaprolactone strands had an average diameter of 360 nm. Variations in the concentration of polycaprolactone had no impact on printability, while variations in the concentration of chitosan did. Although the polycaprolactone composition of the scaffolds may be altered, they nevertheless show considerable potential in terms of swelling, degradation, and mechanical behavior. In vitro, cell adhesion, nontoxicity, low apoptosis, strong proliferation, and biocompatibility of cells have all been shown. The catheter-injectable gelatin-alginate hydrogel expands the scope of tissue engineering and less invasive surgery. It is a low-cost, easy-to-use alternative to the many lung tissue engineering scaffolds now available [103].

This novel method [104] has the potential to be used to develop new regenerative medicine medications. Decellularization and recellularization are two cutting-edge techniques for studying lung regeneration mechanisms that have opened new research possibilities. When native cells are removed from the lungs, a physiological environment favorable to seeded cell regeneration and cutting-edge extracellular matrix research is created. The molecular mechanisms underpinning lung growth and regeneration in mice may be utilized to create techniques of regeneration in larger animals such as humans [105]. Acellular human lung scaffolds may be sliced into small segments, thin slices, or extracellular matrix extracts to study cell behavior such as viability, proliferation, migration, and differentiation. A recent study indicates that sufficient primary endothelium and lung epithelial cells may be produced to recellularize whole lobes, which can then be examined ex vivo in a bioreactor for many days. On the other hand, acellular human lung scaffolds are increasingly being used to study cell–extracellular environment interactions. Acellular human lung scaffolds provide novel in vitro studies of lung repair and regeneration [106]. Organ bioengineering using decellularized whole-organ scaffolds may be a feasible option for addressing donor organ shortages.

**Figure 6:**
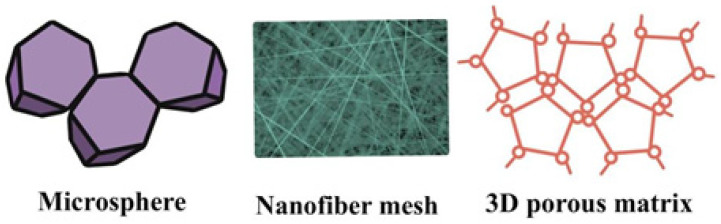
Different types of biopolymeric scaffolds.

On the other hand, recellularizing acellular scaffolds produced from multicellular organs such as the lung remains challenging. Multipotent cells may give birth to cells capable of recellularization. The hierarchical growth of multipotent ES-derived endoderm cells into proximal airway epithelial cells using acellular lung scaffolds has been studied. TP63+/KRT5+ basal cells were the first cells to develop on the scaffolds, followed by multiciliated and secretory airway epithelial cells and, finally, multiciliated and secretory epithelial cells. Basal cells that were TP63+/KRT5+ expressed KRT14 on the scaffolds concurrently with basal cells involved in airway repair after injury. Inhibiting TP63 in ES cells using CRISPR/Cas9 inhibited basal and airway cell development on scaffolds [107]. Graphene is a two-dimensional semiconducting material with enhanced diagnostic sensitivity and response speed, which are essential for COVID-19 spread reduction. Figure 6 shows different types of biopolymeric scaffolds.

To achieve covalent-like stability without interfering with the graphene band structure, docking recognition and antifouling components must be modified. In this study, PA-VS was used to create heterobifunctional supramolecular covalent scaffolds. These scaffolds offer vinylsulfonated awaiting groups for covalent attachment on one side and multivalent pyrene groups on the other. Construction of PA-VS scaffolds was shown using contact angle measurements and Raman spectroscopy [108]. Soft and hard tissue repair and regeneration, as well as wound healing, are possible using electrospun nanofiber scaffolds. Scaffolds work well when the fibers, architecture, and other components are aligned geometrically. The materials (aerogels, microspheres, etc.) and microorganisms used to cure illnesses (cells, proteins, nucleic acids, etc.) have all changed dramatically in terms of adhesion, proliferation, and differentiation. However, scientists must overcome challenges in scaffold design (hydrophilicity, biodegradability, and biocompatibility), as well as commercialization of biological nanofiber products [109]. Descendant pluripotent stem cells and decellularized scaffolds have also been used to construct patient-specific transplantable lungs [110].

### Application Procedure of the Polymer Scaffolds and Polymer Nanoparticles

The presence of cAMP scaffolds and signalosomes in numerous subcellular compartments may induce COPD. In the future, translational research will be required to alleviate illness symptoms by targeting cAMP scaffolds [111]. The scaffolds are made from collagen and collagen analogs using MPE polymerization, which is akin to 3D printing but more precise (0.5 microns). Contrary to expectations, the aligned collagen matrix had a greater impact on these metrics. Notably, these biomimetic lung models cannot be created using standard manufacturing techniques [112]. They were made by electrospinning chitosan, PLA, graphene oxide, titanium dioxide, and doxorubicin composites in chitosan/PLA solutions. They were characterized by XRD, SEM, and TEM. The effect of PLA/chitosan ratio, TiO_2_/DOX content, and GO/TiO_2_/DOX content on DOX release from nanofibrous scaffolds was studied [113]. Gravity seeding with bioreactor culture increased endothelial cell dispersion and engraftment onto rat lung scaffolds. Gravity-driven variable flow perfusion seeds or pumps human umbilical vein endothelial cells into the pulmonary area for seeding with pulsatile flow [114]. Engineered lungs may be created by decellularizing animal lungs and using the extracellular matrix to scaffold human cell recellularization. Eliminating the Gal epitope may help reduce inflammation [115]. Detergent may be beneficial in structuring an acellular lung. Two rat lung scaffolds have been decellularized using SLES and sodium dodecyl sulfate (SDS). SEM, DNA quantification, sulfated GAG quantification, and Western blot were used to evaluate both decellularized lung scaffolds. Later, the scaffolds were placed subcutaneously in rats for six weeks before being removed [116]. Developing viable tissue engineering technologies using decellularized cadaveric or donor lungs may result in a new lung tissue supply. Current lung decellularization treatments employ detergents, and any leftover detergents may affect eventual scaffold recellularization. Detergent removal and quality control processes that thoroughly and consistently validate removal are required to produce acceptable acellular scaffolds for future clinical translation [117]. The ECM of aged animals might cause phenotypic alterations in healthy inoculation cells. Decellularized whole organ scaffolds are a wonderful model for studying ECM-induced cell phenotypes. The influence of age on ECM composition and hLFs has been investigated in native and decellularized rat lungs.

Because RNA is a negatively charged polynucleic acid, it cannot exist in negatively charged membranes. Its thermostability makes it excellent for logic gates, resistive memory, sensor sets, and NEM. Its structure and enzymatic activity are more flexible than proteins [118]. Year-old lungs had fewer laminins 3 and 4, more elastin and fibronectin, and less collagen than 3-week-old lungs [119]. New strategies for expanding cell coverage of vascular surface area and robust manufacture of anticoagulant compounds have been devised. These tactics will be crucial for long-term in vivo function after barrier function is improved. Low-concentration cell suspension has been used, with pulsatile gravity-driven flow, and supraphysiological vascular pressures to seed rat lung microvascular endothelial cells into decellularized rat lungs [120]. The global tissue architecture, essential matrix components, mechanical composition, and seeding capability of lung tissue were all intact [121].

A basic agent-based computer model mimicked cell seeding patterns on a lung scaffold. Mesenchymal stem cells preferentially developed toward the scaffold border, while alveolar epithelial cells did not. This highlights the significance of computational models in understanding cellular activity [122]. In addition, the cell responses to gemcitabine therapy were equivalent to those reported in clinical trials for lung carcinoma, showing that they may help design anticancer treatments by offering more accurate preclinical predictions [123]. PLGA is often used to create porous substrates for complex cell cultures. Substrates or scaffolds with densely packed micrometric pores help build tissue models [124]. Noxious nitric oxide provides antimicrobial action and biofilm dispersion. While no donor scaffolds are currently available, small molecules and macromolecules have been studied [125]. The pore diameter distribution has been studied in microporous systems such as scaffolds, bones, and porous gels. This technique is also used to monitor people with chronic respiratory diseases such as cystic fibrosis [126]. Synthetic polymers are superior in terms of biocompatibility and bioactivity. Their assembly and subsequent engineering allow the construction of innovative hydrogel, fiber, and porous scaffolds. Biopolymeric scaffolds capable of transferring both topographical and electrical inputs have been constructed for mouse neural stem cells [127]. Using aligned electrospun fibers as templates, electrospun fiber-template lithography [128] generates microgroove designs. Biodegradable scaffolds are essential in tissue engineering because they facilitate transient structural regrowth. Synthetic biopolymeric scaffolds have ordered mechanical and biological properties. Personalized patient-specific scaffolds with complex architecture are already conceivable [129]. Synthetic acellular poly (glycerol sebacate) and natural gelatin biopolymeric scaffold sponges may infiltrate and remodel cells [130].

## 5. Discussion

Current treatment for COVID-19 disease involves the administration of several medicines, including anti-viral, anti-malarial, and anti-inflammatory therapies, all of which are currently under investigation. While these therapies may aid inpatient rehabilitation and survival, they do not appear to be effective in restoring the lung damage caused by this disease to lungs’ pre-illness state, according to current research. Embryonic stem cell therapies, and more recently, their released EVs, are emerging as new and potential treatments for COVID-19-induced lung damage, according to the National Institutes of Health. It is possible that these treatments will not only help to decrease inflammation, but they may also help to heal lung damage.

Most likely, EVs are responsible for the therapeutic activities of stem cells, which include immunomodulatory, antioxidant, and reparative properties. As a result, stem cells may be beneficial in the treatment of patients, either alone or in combination with other treatment drugs. As a result of infection in stem cells, there is the possibility of a decrease in regeneration capacity, which may contribute to the severity of SARS-CoV-2 infection as well as the consequences of the infection. The severe consequences of ARDS have been identified. SARS-CoV-2 is a virus that belongs to the coronaviridae family and is responsible for the transmission of respiratory illnesses such as swine flu.

Many inflammatory cytokines are produced because of an infection, resulting in a phenomenon known as a cytokine storm. As a result of the inflammation caused by these cytokines, lung damage may occur, which can result in ARDS and, in some instances, death. New biological materials are needed and scaffolds so that more precise research can be carried out while also assisting in the regeneration of lung tissue to avoid or reduce the mortality associated with lung diseases. The outbreak of the COVID-19 virus, which is particularly targeted at the human lung, has resulted in a significant number of deaths all over the world. Several off-label medicines that have previously been authorized for use in other diseases are now being evaluated in several clinical studies that are currently under way. A wide range of pathologic illnesses, including acute and chronic lung diseases, as well as their therapy using MSCs, have been investigated in animal models and in people over the past two decades.

A brief explanation of biopolymeric nanoparticle methods and roles in tissue regeneration is shown in Table 1. Furthermore, a brief explanation of inorganic nanoparticle methods and roles in tissue regeneration is shown in Table 2. Clinical studies of experimental treatment for ARDS, the most severe consequence of COVID-19 infection, have shown promising outcomes in most cases. Approximately 90% of clinical treatment is supportive in nature and is dependent on the patient’s immune response, which may result in an immune reaction storm, lung edema, airway dysfunction, and ARDS, all of which can lead to multiorgan failure and death. When enough primary endothelium and lung epithelial cells are produced, they may be utilized to decellularize whole lobes of the lung, which can then be examined ex vivo in a bioreactor for many days at a time. Acellular human lung scaffolds, on the other hand, are increasingly being used in the laboratory to study the interactions between cells and their surrounding extracellular environment. It is certain that more cooperation and the development of new technologies are required in current research to accelerate the process of lung tissue regeneration and regrowth.

## 6. Conclusions

A new generation of medications is urgently required to counterbalance the hyperactive immune response, retain alveolar function, and cure lung and systemic organ damage resulting from COVID-19. Poor lung circulation, scarring, and airway occlusion have been linked to COVID-19 exposure. A lack of oxygen in the blood was a major concern for many researchers, and they wanted to find a scientific method for reviving those cells as rapidly as possible so that the patient would not have trouble with other bodily systems. Herein, the most recent biopolymeric particle-based technologies and approaches have been presented to show their important roles in lung tissue regeneration. The main topics that have been presented in this review are the use of biopolymeric nanoparticles, stem cell materials, and biopolymeric scaffolds. Their roles in regenerating lung tissues have been clarified considering their functionalities, structures, and merging with other types of particles. The various methods and processes for regenerating pulmonary tissue have been thoroughly investigated. A growing number of treatments for regenerating lung tissue emerged throughout the inquiry. Further research is obviously required to speed up the regeneration of lung tissue.

## 7. Future Perspectives

The functionalities of biopolymer particles may open new avenues for polymer and surface scientists to make significant contributions to lung tissue regeneration soon. In the future, natural-derived biopolymers should be employed for 3D printing to provide a more accurate representation of the structure of epithelial cells. More efforts should be made to utilize lung gene therapy in the treatment of lung disease. Due to a variety of obstacles, including the lack of an adequate vector, lung gene therapy for cystic fibrosis illness has proved unsuccessful so far. As a result, non-viral delivery mechanisms that are appropriate for the administration of developing therapies to airway epithelial cells are required for effective delivery. In addition, it is required to limit the use of biopolymers as surgical implants in the pulmonary blood vessel system. Furthermore, it is anticipated that biopolymer particles will play a significant role in the recovery from severe chronic inflammation, deadly lung fibrosis, and cell death, among other conditions. It is envisaged that the processes by which mucin biopolymers protect the lung from harm will be discovered because of the continuous advancement of nanodevices and nanocarrier production technology. Drugs may soon be entrapped in a polymer-based cavity or adsorbed into the lung tissues, which would represent a significant advancement in the treatment of inflammation in infected cells.

## Figures and Tables

**Table 1:** Biopolymeric nanoparticle methods and roles in tissue regeneration.

Nanoparticle Types	Methods/Roles	Ref.
PCL	Electrospinning of PCL and ZnO particles creates the membranes	[54]
LNPs	employed to carry plasmid DNA into the nucleus of host cells in lung tissues	[55]
PLGA	used to create porous substrates for complex cell cultures	[124]
TT3	nanoparticles enhance the expression of putative SARS-CoV-2 antigens	[52]
MC3	elicits almost two orders of magnitude more antigen-specific antibodies	[52]
UCNPs	deterioration of the hydrogels was assessed utilizing a simple and non-invasive in vivo hydrogel tracking methodology	[27]
LPH	boost the medicine’s efficacy	[35]
PLA	PLA/chitosan ratio, TiO_2_/DOX content, and GO/TiO_2_/DOX content influence DOX release from nanofibrous scaffolds	[113]
TW-37	TW-37, a novel KIM-1-mediated endocytosis inhibitor, and anti-Kim-1 antibodies both reduced absorptions	[33]
Nanocurcumin	therapeutic effects on the quantity and reactivity of Th17 cells in COVID-19 patients	[48]

**Table 2:** Inorganic nanoparticle methods and roles in tissue regeneration.

Nanoparticle Types	Methods/Roles	Ref.
IONPs	Interaction of magnetic IONPs (Fe_2_O_3_ and Fe_3_O_4_) with the SARS-CoV-2 spike protein	[49]
PER NPs	MOMCs have many PER NPs on their surface that target the lungs	[26]
LIONs	The LION/repRNA-CoV2S vaccination elicited anti-SARS-CoV-2 S protein IgG antibody isotypes suggestive of a type 1 T helper cell response	[31]
ZnO	interaction of ZnO NPs with COVID-19 targets such as the ACE2 receptor	[32]
AgNP	are often used to treat acute and chronic wounds	[57]
AuNPs	potential to accelerate wound healing by stimulating tissue regeneration, connective tissue development, and angiogenesis	[58]
anti-CoV NPs	addresses nano–bio interactions, in vitro (lung cells), and in vivo assessments, as well as human ramifications	[39]

## Data Availability

The data presented in this study are available within the article.

## Abbreviations

COVID-19	coronavirus disease 2019
IONPs	magnetic iron oxide nanoparticles
Fe_2_O_3_	ferric oxide
Fe_3_O_4_	ferrosoferric oxide
SARS-CoV-2	severe acute respiratory syndrome coronavirus 2
RDB	receptor binding domain
NPs	nanoparticles
MOI	multiplicities of infection
MSCs	mesenchymal stem cells
RNA	ribonucleic acid
NEM	Nanoelectromechanical
DNA	deoxyribonucleic acid
NRP1	neuropilin-1
DCs	dendritic cells
LNPs	lipid nanoparticles
HPD	hesperidin
ACE2	angiotensin-converting enzyme 2
LPS	lipopolysaccharide
ARDS	acute respiratory distress syndrome
qRT-PCR	quantitative real-time polymerase chain reaction
AL-Rem	remdesivir
Calu-3	submucosal gland cell line
PLGA	poly (lactic-co-glycolic acid)
RdRp	RNA-dependent RNA polymerase
NASAR	novo design of UTRs, the optimal combination of 5′ and 3′ UTR
TT3	total triiodothyronine
FDA	Food and Drug Administration
MC3	composition of composite material 3
LOAC	lung-on-a-chip
NIR-II	second near infrared
CT	computed tomography
GLCM	gray-level co-occurrence matrix
hACE2	human angiotensin I-converting enzyme 2
IPF	idiopathic pulmonary fibrosis
AEC II	type II alveolar epithelial cells
PER NPs	surface-engineered nanoparticles
MOMC	monocyte-derived multipotent cells
AST	astaxanthin
TRA	trametinib
MMP-2	matrix metalloproteinase 2
UCNPs	upconverting nanoparticles
FITC	fluorescein isothiocyanate
LCSTs	lower critical solution temperatures
MERS-CoV	Middle East respiratory syndrome coronavirus
H1N1	swine flu
LIONs	lipid inorganic nanoparticles
IgG	immunoglobulin G
ZnO	zinc oxide
KIM-1	kidney injury molecule-1
miRNAs	MicroRNAs
LPH	lipid polymer hybrid
SSRIs	selective serotonin reuptake inhibitors
FH	fluoxetine hydrochloride
hsACE2	a soluble form of hACE2
MHRA	Medicines and Healthcare Products Regulatory Agency
ABL-2	Abelson-related gene
IL-1beta	interleukin-1-beta
IL-6	interleukin 6
IL-8	interleukin-8
Mip-1alpha	macrophage inflammatory protein-1 alpha
TNF-alpha	tumor necrosis factor alpha
PI4Kiiibeta	PI4KB phosphatidylinositol 4-kinase beta
IBD	inflammatory bowel disease
AlphaMSH	alpha-melanocyte-stimulating hormone
MC1R	melanocortin 1 receptor
NO	nitric oxide
AFM	atomic force microscopy
ROS	reactive oxygen species
CCL2	C–C motif chemokine ligand 2
Th17	T helper 17
IL-6	interleukin-6
IL-17	interleukin-17
IL-18	interleukin-18
IL-21	interleukin-21
IL-23	interleukin-23
EVs	extracellular vesicles
SARDS	severe acute respiratory distress syndrome
ALI	acute lung injury
ASCs	adipose stem cells
ICU	intensive care unit
FiO_2_	fraction of inspired oxygen
SaO_2_	oxygen saturation
PO_2_	partial pressure of oxygen
LSCs	lung stem cells
7nAChR	vagal-alpha7 nicotinic acetylcholine receptor
MSCs-Exo	mesenchymal stem cells and their exosomes
BASCs	bronchioalveolar stem cells
ALOs	adult lung organoids
hiPSC-CMs	human induced pluripotent stem cell-derived cardiomyocytes
CDC42	cell division cycle 42
TGF	transforming growth factor
iAT2s	alveolar type 2-like cells
UC-MSCs	umbilical cord-mesenchymal stem cells
P (VDF-TrFE)	poly (vinylidene fluoride-co-trifluoroethylene)
HBD2	human beta defensin 2
MRC-5	Medical Research Council cell strain 5
iPSCs	induced pluripotent stem cells
COPD	chronic obstructive pulmonary disease
cAMP	cyclic adenosine monophosphate
MPE	multiphoton excited
IPF	idiopathic pulmonary fibrosis
PLA	polylactic acid or polylactide
GO	graphene oxide
DOX	doxorubicin
TiO_2_	titanium dioxide
XRD	X-ray powder diffraction
SEM	scanning electron microscopy
TEM	transmission electron microscopy
SLES	sodium lauryl ether sulfate
SDS	sodium dodecyl sulfate
GAGs	glycosaminoglycans
ECM	extracellular matrix
hBECs	human bronchial epithelial cells
hLFs	lung fibroblasts
NETs	neutrophil extracellular traps
MPO	myeloperoxidase
PCL	poly(caprolactone)
AgNP	silver nanoparticle
AuNPs	gold nanoparticle
hAFS	human amniotic fluid stem
ToF-SIMS	time-of-flight secondary ion mass spectrometry
PA-VS	vinyl-sulfonated polyamines
